# Embolization of Ruptured Aneurysms in the Intracranial Peripheral Arteries Using N-Butyl Cyanoacrylate Glue: A Case Series

**DOI:** 10.7759/cureus.71028

**Published:** 2024-10-07

**Authors:** Yosuke Sakai, Tetsuya Tsukada, Toru Watanabe, Yoshio Araki, Yukio Seki

**Affiliations:** 1 Neurosurgery, Japanese Red Cross Aichi Medical Center Nagoya Daini Hospital, Nagoya, JPN

**Keywords:** endovascular, glue embolization, n-butyl cyanoacrylate glue, parent artery occlusion, peripheral aneurysm

## Abstract

Ruptured aneurysms in peripheral arteries are rare, and an optimal treatment strategy has not yet been established. Herein, we evaluated the efficacy and safety of endovascular treatment of peripheral aneurysms located in small, tortuous vessels. In the present study, we report five consecutive cases of small, peripherally located ruptured aneurysms successfully embolized using N-butyl cyanoacrylate glue, with or without the adjunct use of coils. Five ruptured fusiform peripheral artery aneurysms in five consecutive patients were treated with parent artery occlusion using N-butyl cyanoacrylate glue. The etiologies included a flow-related feeder aneurysm associated with cerebral arteriovenous malformation in one case, a mycotic aneurysm associated with infective endocarditis in one case, and unknown causes in three cases. The procedural safety was assessed in advance using a provocation test in combination with the test occlusion of the parent artery with a coil, as required. Complete occlusion of the aneurysm was achieved in all patients, and no instances of postoperative symptomatic cerebral infarction were observed. The patients who attended follow-up showed no recurrence of aneurysms. This study shows that small peripheral artery aneurysms can be effectively embolized endovascularly with high therapeutic efficacy, using an appropriately sized microcatheter, after conducting a thorough safety assessment of parent artery occlusion.

## Introduction

Intracranial peripheral aneurysms are rare and typically form secondary to infection, inflammation, traumatic injury, or growth associated with arteriovenous malformations (AVMs) or Moyamoya disease [1,2]. However, recent reports have described cases of peripherally located small aneurysms of unknown etiology [3]. Treating these small aneurysms in the peripheral arteries presents significant challenges due to the difficulty in identifying lesions during craniotomy, as well as the complexities of guiding a microcatheter into small, tortuous peripheral arteries during endovascular procedures [1,4]. Advances in catheter design have allowed for peripheral aneurysms to be reached, providing an advantage for endovascular treatment and increasing treatment options [5,6].

In this study, we present five consecutive cases of small, ruptured, peripherally located aneurysms successfully embolized using N-butyl cyanoacrylate (NBCA) glue, with or without the adjunct use of coils. We evaluated the efficacy and safety of this technique. The study discusses the importance of catheter selection according to the parent artery diameter.

## Case presentation

This study enrolled five peripherally located aneurysms in five consecutive patients treated between August 2022 and March 2023, utilizing parent artery occlusion with NBCA. The aneurysm onset type was subarachnoid hemorrhage (SAH) in three cases and intracerebral hemorrhage (ICH) in two cases. Diagnosis was confirmed through digital subtraction angiography (DSA), using the ARTIS icono D-Spin (Siemens Healthineers, Tokyo, Japan) (Table 1). The aneurysm locations were classified according to the past literature [7].

**Table 1:** Summary of peripherally located aneurysms treated by embolization ACA: anterior cerebral artery, AVM: arteriovenous malformation, ICH: intracerebral hemorrhage, IE: infective endocarditis, LSA: lenticulostriate artery, MCA: middle cerebral artery, NBCA: N-butyl cyanoacrylate glue, PICA: posteroinferior cerebellar artery, SAH: subarachnoid hemorrhage

Case No.	Age (years)	Sex	Type of Onset	Aneurysm Location	Parent Artery Size (mm)	Aneurysm Size (mm)	Etiology of Aneurysm	Intraoperative Anesthesia	Microcatheter	Distal Access Catheter	Provocation Test	Embolization Materials
1	38	M	ICH	Lt. ACA A5	0.5	1.2	unknown	local and intravenous	DeFrictor Nano	Guidepost	Yes	NBCA
2	68	F	SAH	Lt. MCA M4	0.4	1.0	unknown	local and intravenous	DeFrictor Nano	Guidepost	Yes	NBCA
3	59	M	SAH	Lt. MCA M4	0.9	5.0	mycotic aneurysm associated with IE	local and intravenous	Marathon	Guidepost	Yes	NBCA and Coil
4	64	M	ICH	Lt. LSA	0.4	1.2	feeder aneurysm associated with AVM	general	DeFrictor Nano	Guidepost	No	NBCA
5	78	F	SAH	Lt. PICA inferior vermian branch	0.5	1.6	unknown	local and intravenous	Marathon	Guidepost	No	NBCA

All aneurysms were fusiform, with sizes ranging from 1.0 to 5.0 mm and parent artery diameters ranging from 0.4 to 0.9 mm. Four of the five aneurysms were microaneurysms, with sizes of 1.0 to 1.6 mm, and parent artery diameters sized between 0.4 and 0.5 mm. The etiologies included a flow-related feeder aneurysm associated with a cerebral AVM in one case, a mycotic aneurysm associated with infective endocarditis in one case, and unknown causes in three cases.

In three cases, a provocation test was performed prior to embolization to evaluate the risk of procedure-related symptomatic ischemic complications. The test was skipped in two cases due to poor consciousness in one patient and the absence of normal brain tissue supplied by the feeding artery in the other case. Four aneurysms were embolized using NBCA alone, and one was embolized using both NBCA and coils. In all cases, complete aneurysm occlusion was achieved without any symptomatic ischemic complications.

Case 1

**Figure 1:**
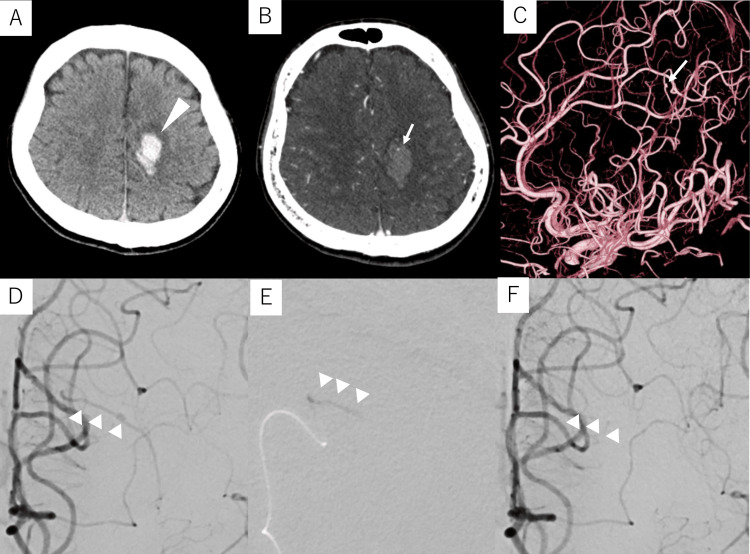
Preoperative and intraoperative imaging of Case 1 Case 1: A 38-year-old male with intracerebral hemorrhage (A) Computed tomography (CT) upon admission shows a left frontal subcortical hemorrhage (arrowhead). (B) Contrast-enhanced CT shows an enhanced spot (arrow) within the hematoma. (C, D) Digital subtraction angiography (DSA) of the left internal carotid artery shows a 1.1-mm fusiform aneurysm (arrow) on a 0.5-mm peripheral branch (arrowheads) of the left anterior cerebral artery. (E) The aneurysm and the parent artery are embolized with 33% N-butyl cyanoacrylate glue (arrowheads). (F) Post-embolization DSA of the left internal carotid artery shows the disappearance of the aneurysm (arrowheads).

A 38-year-old male with no prior health issues who experienced sudden-onset right-leg paralysis was transferred from a local physician to our hospital with a diagnosis of ICH. Computed tomography (CT) revealed a left frontal subcortical hemorrhage (Figure 1A) while contrast-enhanced CT identified an enhanced spot suggestive of a bleeding source within the hematoma (Figure 1B). DSA revealed a 1.1-mm fusiform aneurysm on a 0.5-mm peripheral branch of the left anterior cerebral artery (ACA), indicating the need for NBCA embolization of the lesion (Figures 1C, 1D). Under local anesthesia, a 6-Fr long sheath was inserted into the right femoral artery. A 6-Fr Slim Guide^®^ (Medikit, Tokyo, Japan) was subsequently introduced into the left internal carotid artery (ICA), through which a Guidepost (Tokai Medical Products, Aichi, Japan) was positioned at the A1 portion of the left ACA as a distal access catheter (DAC). A DeFrictor Nano microcatheter (Medico’s Hirata, Osaka, Japan) was subsequently navigated into the parent artery of the aneurysm using a CHIKAI X10 guidewire (ASAHI INTECC, Tokyo, Japan). As the provocation test with 1% lidocaine did not result in any worsening of paralysis or the appearance of Gerstmann’s symptoms, the aneurysm and parent artery were completely embolized with 33% NBCA (Figures 1E, 1F).

The patient’s postoperative course was uneventful, and he was transferred to a rehabilitation hospital on postoperative day (POD) 13. At the three-month postoperative follow-up, the patient showed significant recovery and was able to run. Additionally, DSA performed five months after embolization revealed no recanalization of the aneurysm.

Case 2

**Figure 2:**
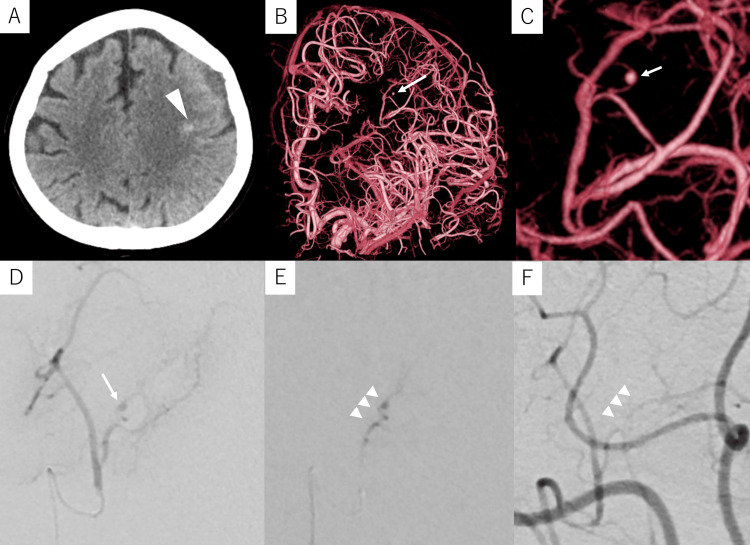
Preoperative and intraoperative imaging of case 2 Case 2: A 68-year-old woman with a small subarachnoid hemorrhage. (A) Computed tomography upon admission shows a small amount of subarachnoid hemorrhage in the left frontal area (arrowhead). (B, C) Digital subtraction angiography (DSA) of the left internal carotid artery shows a 1.0-mm fusiform aneurysm (arrow) on a peripheral branch of the left middle cerebral artery. (D) Selective angiography of the parent artery (working-angle view) shows an aneurysm (arrow). (E) The aneurysm and parent artery are embolized with 33% N-butyl cyanoacrylate glue (arrowheads). (F) Post-embolization DSA of the left internal carotid artery shows the disappearance of the aneurysm (arrowheads).

A 68-year-old woman with a medical history of colorectal cancer presented to a local physician with dysarthria. Head CT revealed a small SAH in the left frontal area, and the patient was subsequently referred to our hospital (Figure 2A). Contrast-enhanced CT revealed no aneurysms or contrast lesions within the hematoma. DSA of the left ICA revealed a 1.0-mm fusiform aneurysm on a peripheral branch (0.4 mm in diameter) of the middle cerebral artery (MCA) (Figures 2B-2D), indicating the need for NBCA embolization. Under local anesthesia, a 6-Fr ASAHI FUBUKI (ASAHI INTECC) was inserted into the left femoral artery and advanced into the left ICA. A Guidepost was subsequently placed in the M1 portion of the left MCA as a DAC, and a DeFrictor^®^ Nano microcatheter (Medico’s Hirata) was introduced into the parent artery using the CHIKAI X10 guidewire (ASAHI INTECC). As the provocation test with 1% lidocaine was negative, the aneurysm was completely embolized with 33% NBCA (Figures 2E, 2F).

The patient’s postoperative course was uneventful, and DSA performed on POD 8 revealed no recanalization of the aneurysm. The patient was discharged on POD 10. No recurrence was noted in magnetic resonance imaging at the one-year follow-up.

Case 3

**Figure 3:**
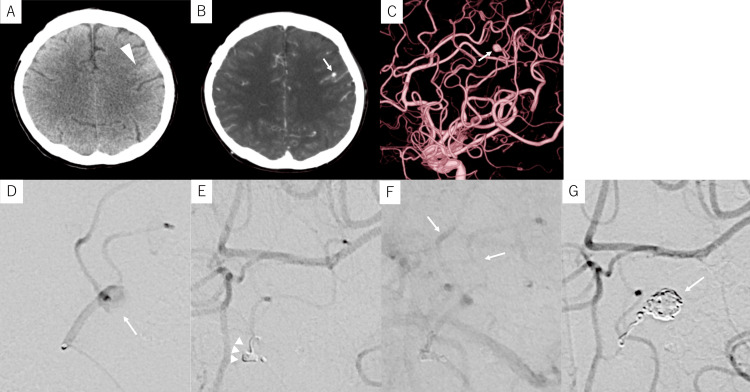
Preoperative and intraoperative imaging of Case 3 Case 3: A 59-year-old man with a small subarachnoid hemorrhage. (A) Computed tomography (CT) upon admission shows a small amount of subarachnoid hemorrhage in the left frontal sulcus (arrowhead). (B) Contrast-enhanced CT shows a small enhanced lesion (arrow) within the hematoma. (C) Digital subtraction angiography (DSA) of the left internal carotid artery (lateral view) shows a 5.0-mm fusiform aneurysm (arrow) on the peripheral branch of the left middle cerebral artery. (D) Selective angiography of the parent artery (working-angle view) shows a fusiform aneurysm (arrow). (E, F) DSA of the left internal carotid artery while performing an occlusion test of the parent artery with a coil (arrowheads): (E) Occlusion of the parent artery in the arterial phase; (F) Delayed filling downstream (arrows) in the venous phase. (G) Post-embolization DSA of the left internal carotid artery shows complete occlusion of the aneurysm (arrow).

A 59-year-old man with a medical history of hypertension, hyperlipidemia, or previous clipping of a ruptured aneurysm in the right MCA M2 segment presented to our hospital with mild right-hand weakness and dysarthria. Head CT revealed a small SAH in the left frontal sulcus (Figure 3A) while contrast-enhanced CT identified a small, enhanced lesion within the sulcus, suspected to be an aneurysm (Figure 3B). The patient had lost 5 kg of body weight over the preceding six months and experienced persistent fever for two months prior to onset. Blood tests revealed an elevated inflammatory response while echocardiography showed vegetation on the mitral valve. Based on these findings, bleeding from an infected aneurysm due to infective endocarditis was suspected. DSA revealed five aneurysms, including a 5.0-mm fusiform aneurysm on a peripheral branch (0.9 mm in diameter) of the left MCA at the site of bleeding (Figures 3C, 3D). Under local anesthesia, an 8-Fr Optimo (Tokai Medical Products) was guided to the left ICA, and a Guidepost was placed in the M2 portion of the left MCA as a DAC. A Marathon microcatheter (eV3; Covidien, Irvine, CA, USA) was then navigated to the parent artery using a Tenrou 1014 guidewire (Kaneka Medics, Osaka, Japan). Since the provocation test with 1% lidocaine elicited temporary paralysis of the right face and upper limb, a parent artery test occlusion with a 0.010-inch coil (i-ED COIL Complex Silky Soft, 1.5 mm × 3 cm; Kaneka Medics, Osaka, Japan) was performed. This test was negative, with radiological evidence of retrograde opacification of the peripheral part of the artery during the test (Figures 3E, 3F), indicating that embolization of the selected artery segment was safe. As the embolization of both the aneurysm and parent artery with seven coils was incomplete, 0.01 ml of 50% NBCA was added to achieve complete obliteration of the lesion (Figure 3G).

DSA performed on postoperative day (POD) 6 revealed no recanalization of the aneurysm. After receiving four weeks of antibiotic treatment, the patient was discharged without any new neurological symptoms. No recurrence was noted in DSA at the one-year follow-up.

Case 4

**Figure 4:**
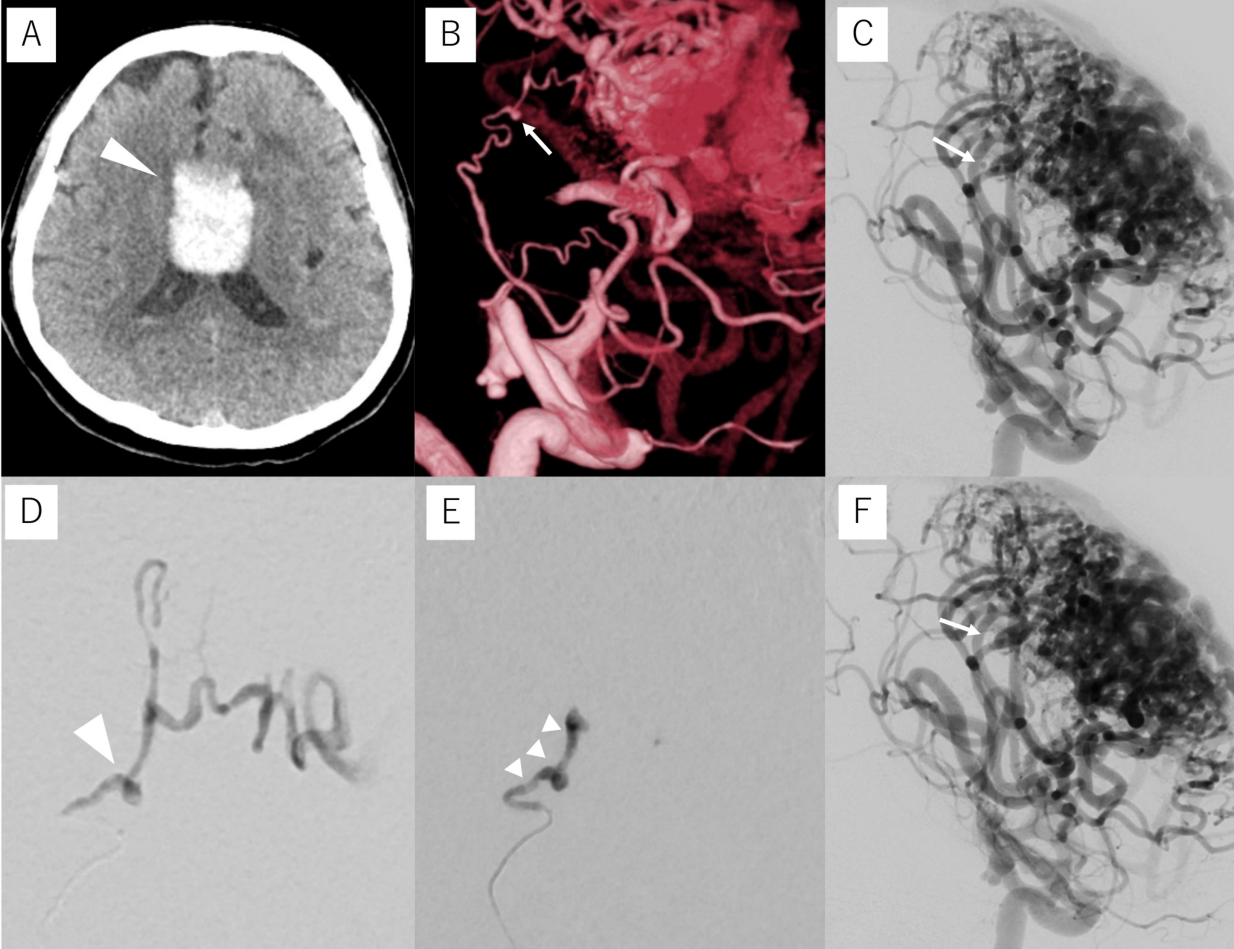
Preoperative and intraoperative imaging of Case 4 Case 4: A 64-year-old male with an intracerebral hemorrhage near the corpus callosum. (A) Computed tomography (CT) upon admission shows a cerebral hemorrhage near the corpus callosum (arrowhead). (B, C) Digital subtraction angiography (DSA) of the left internal carotid artery shows AVM and a 1.2-mm fusiform aneurysm (arrow) on a lenticulostriate artery. (D) Selective angiography of the parent artery (working-angle view) shows a fusiform aneurysm (arrowhead). (E) The aneurysm and parent artery are embolized with 33% N-butyl cyanoacrylate glue (arrowheads). (F) Post-embolization DSA of the left internal carotid artery shows the disappearance of the aneurysm (arrow).

A 64-year-old male with a history of untreated AVM was transferred to our hospital with mild right upper and lower paralysis and dysarthria. Head CT revealed an ICH near the corpus callosum (Figure 4A) while DSA showed a 1.2 mm microaneurysm in a lenticulostriate artery with a diameter of 0.4 mm (Figures 4B-4D); subsequently, we decided to perform TAE with NBCA under general anesthesia. A 9Fr long sheath was inserted into the right FA, and a 9Fr Optimo was guided to the left ICA. As in the previous case, a Guidepost was used as a DAC, and a Defrictor Nano microcatheter (Medico’s Hirata) was guided to the vicinity of the aneurysm. The aneurysm was completely embolized with 0.01 ml of 33% NBCA (Figures 4E, 4F). The patient was transferred to a rehabilitation hospital without any postoperative worsening of paralysis. No recurrence was noted on DSA at the six-month follow-up.

Case 5

**Figure 5:**
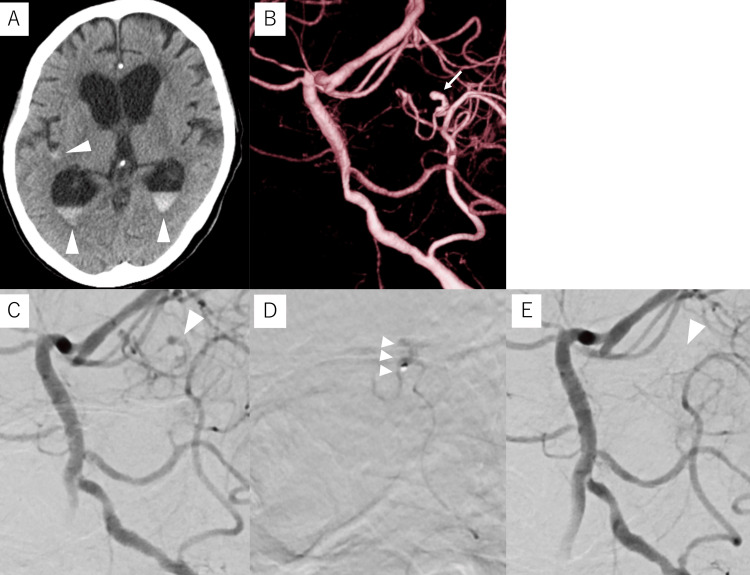
Preoperative and intraoperative imaging of Case 5 Case 5: A 78-year-old female with intraventricular hemorrhage and subarachnoid hemorrhage in the Sylvian fissure. (A) Computed tomography (CT) upon admission shows an intraventricular hemorrhage and subarachnoid hemorrhage in the Sylvian fissure (arrowheads). (B) Digital subtraction angiography (DSA) of the left vertebral artery shows a 1.6-mm fusiform aneurysm (arrow) on an inferior vermian branch of the posterior inferior cerebellar artery. (C) Selective angiography of the parent artery (working-angle view) shows a fusiform aneurysm (arrowhead). (D) The aneurysm and parent artery are embolized with 20% N-butyl cyanoacrylate glue (arrowheads). (E) Post-embolization DSA of the left vertebral artery shows the disappearance of the aneurysm (arrowhead).

A 78-year-old female, institutionalized with dementia, was transferred to our hospital due to consciousness disorder. Head CT revealed intraventricular hemorrhage and SAH in the Sylvian fissure (Figure 5A). DSA revealed a 1.6 mm microaneurysm in a 0.5 mm vermian branch of the posterior inferior cerebellar artery (Figures 5B, 5C). We decided to perform TAE with NBCA under local anesthesia. A 6Fr long sheath was inserted, and a 5Fr Slim guide^®^ (Medikit, Tokyo, Japan) was guided to the left vertebral artery. As in the previous case, a Marathon microcatheter was guided to the vicinity of the aneurysm. The aneurysm was completely embolized with 0.01 ml of 20% NBCA (Figures 5D, 5E). Postoperative MRI revealed no obvious infarction, and the patient improved to the point of communication. The patient was discharged to the facility with a modified Rankin Scale (mRS) of 4, after which subsequent follow-up could not be performed.

## Discussion

Herein, we report five cases of small, ruptured, peripherally located aneurysms successfully embolized using NBCA. All patients who underwent follow-up showed no evidence of postoperative symptomatic cerebral infarction and no aneurysm recurrence after six months to one year of follow-up for patients.

Intracranial peripheral aneurysms are rare [8], accounting for approximately 7-9% of ACA aneurysms, 2-7% of MCA aneurysms, and 5% of posterior cerebral artery aneurysms [4]. Most aneurysms result from etiologies such as infection, inflammation, and traumatic injury [1,2]. However, there have also been several reports of peripheral aneurysms with no apparent etiology [3]. Recent advances in diagnostic imaging tools with improved spatial resolution may have contributed to the increased detection of small, peripherally located aneurysms, similar to those observed in our cases [9]. In cases where a contrast-enhanced area is detected within the hematoma, such as those in the present series, or in cases of SAH where no aneurysm is detected on contrast-enhanced CT, further close examination with DSA is required to assess the possibility of microaneurysms. Microaneurysms are relatively small, have a high rupture rate, are often associated with intracranial hemorrhage, and are associated with significant morbidity and mortality [1]. In a previous report, Isokangas et al. reported that three out of seven ruptured peripheral aneurysms of the posterior inferior cerebellar artery re-ruptured early prior to endovascular treatment [10]. Although guidelines for managing intracranial peripheral aneurysms that are too small to embolize with coils alone have not yet been established, endovascular parent artery occlusion with liquid embolic materials appears to be a preferable procedure, particularly when these aneurysms are not associated with large hematomas requiring urgent craniotomy [4,10-13].

While recent studies have reported on the treatment of intracranial peripheral aneurysms [12-14], few have assessed the diameter of the parent artery or described specific treatment strategies such as catheter selection. The process of guiding a microcatheter into the peripheral arteries can be challenging due to the risk of intraoperative hemorrhage, particularly in small, tortuous arteries. In this case series, all aneurysms were located in small tortuous arteries, with diameters of less than 1 mm, including four aneurysms located in parent arteries with diameters of less than 0.5 mm. This makes the choice of microcatheter crucial to ensure successful endovascular treatment.

For slightly larger parent arteries, a Marathon microcatheter (eV3) with a distal-end outer diameter of 1.5 Fr was selected due to its ability to deliver coils. This choice allowed for both temporary test occlusion of the parent arteries and aneurysm occlusion. In cases involving very small parent arteries, a thin and flexible DeFrictor® Nano catheter (Medico’s Hirata) with a distal-end outer diameter of 1.3 Fr was selected. This catheter allowed us to approach the aneurysm closely, minimizing the area of arterial embolization, improving control of NBCA by wedging the parent artery near the aneurysm, and preventing the unintended dispersion of the embolic material. In all cases, the use of a DAC was beneficial for enhancing microcatheter manipulation in tortuous peripheral arteries. As such, we believe that appropriate microcatheter selection based on the size of the parent artery is essential for ensuring safer and more effective parent artery occlusion.

Ischemia in the arterial territory is the major complication associated with parent artery occlusion. This post-procedural symptomatic ischemia is typically predicted using super-selective amytal provocation testing or balloon test occlusion [13,15,16]. However, Eckard et al. previously suggested the possibility of overestimating ischemia using the provocation test due to drug permeation into surrounding vessels and potential compensation for ischemia after parent artery occlusion by leptomeningeal collateral blood supply [13]. Douds et al. further reported the functional significance of parent artery test occlusion using a Guglielmi detachable coil (Boston Scientific, Marlborough, Massachusetts, US) finding no worsening of neurological symptoms during test occlusion and safely achieving parent artery occlusion [17]. Therefore, in the present series, we employed the same technique in one case in which the lidocaine provocation test was positive. Based on the negative result using this technique, the parent artery was occluded without causing symptomatic brain infarction. Notably, the provocation test must be performed under local anesthesia to confirm neurological symptoms, necessitating frequent repeat angiography and careful catheter guidance to mitigate the risks of vascular perforation and discrepancies between the road map and the actual vascular course due to patient movement.

This study has some limitations, including the small number of cases and single-institution design. To establish parent artery embolization as a reliable treatment for small, peripherally located aneurysms, further investigations in a larger cohort will be required. Additionally, the nature of small peripheral aneurysms without a known etiology needs to be elucidated.

## Conclusions

Overall, this case series showed that ruptured aneurysms in intracranial peripheral arteries can be effectively treated by parent artery occlusion with NBCA. Complete occlusion of the aneurysm was obtained in all patients in this study, with no symptomatic ischemic complications or aneurysm recurrence at least at the one-year follow-up. In NBCA embolization, microcatheters should be selected according to the parent artery diameter and whether or not a coil is used in conjunction.
